# Determining lines of therapy in patients with solid cancers: a proposed new systematic and comprehensive framework

**DOI:** 10.1038/s41416-021-01319-8

**Published:** 2021-04-13

**Authors:** Kamal S. Saini, Chris Twelves

**Affiliations:** 1 Covance Inc., Princeton, NJ USA; 2 East Suffolk and North Essex NHS Foundation Trust, Ipswich, UK; 3 University of Leeds and Leeds Teaching Hospitals Trust, Leeds, UK

**Keywords:** Chemotherapy, Drug development

## Abstract

The complexity of neoplasia and its treatment are a challenge to the formulation of general criteria that are applicable across solid cancers. Determining the number of prior lines of therapy (LoT) is critically important for optimising future treatment, conducting medication audits, and assessing eligibility for clinical trial enrolment. Currently, however, no accepted set of criteria or definitions exists to enumerate LoT. In this article, we seek to open a dialogue to address this challenge by proposing a systematic and comprehensive framework to determine LoT uniformly across solid malignancies. First, key terms, including LoT and ‘clinical progression of disease’ are defined. Next, we clarify which therapies should be assigned a LoT, and why. Finally, we propose reporting LoT in a novel and standardised format as LoT N (CLoT + PLoT), where CLoT is the number of systemic anti-cancer therapies (SACT) administered with curative intent and/or in the early setting, PLoT is the number of SACT given with palliative intent and/or in the advanced setting, and N is the sum of CLoT and PLoT. As a next step, the cancer research community should develop and adopt standardised guidelines for enumerating LoT in a uniform manner.

## Background

The treatment of solid cancers typically involves the use of multiple modalities, such as surgery, systemic anti-cancer therapy (SACT) and radiotherapy, alone or in combination or sequentially. The increasing efficacy of anti-cancer therapy has resulted in improved clinical outcomes, and it is not uncommon for a patient with an advanced malignancy to have received successive SACTs, often over the course of many years. Furthermore, several different types of SACT (e.g. cytotoxic, endocrine therapy, targeted therapy, immunotherapy and so on) are available, and are used in different settings (e.g. adjuvant, neoadjuvant, locally advanced and palliative). Standard treatment guidelines for patients with advanced cancer might be lacking or non-existent, and these patients can receive an enormous range of different treatments. The resulting clinical pathways of real-life patients are often, therefore, far more complex than those defined by standard guidelines, expert opinion, consensus discussions and clinical trials and, indeed, very few patients experience the idealised patient pathway that is initially outlined in their care plan.^1^

The accurate enumeration of prior anti-cancer therapies is critically important for a number of reasons: selecting optimal future treatment for individual patients;^2^ conducting effective medication and clinical auditing;^3^ determining eligibility for enrolment into clinical trials; facilitating adherence to approved indications; enabling more accurate health technology assessment; and for the purposes of re-imbursement.^4^ In the era of evidence-based medicine, it is important that prior lines of therapy (LoT) be defined and counted in a manner that is consistent, rational and comprehensive.

Currently, most trials and researchers refer to LoT using a single ordinal number—for example, ‘first-line therapy’—often applied in imprecise, confusing and conflicting ways. In the oncology literature, the term ‘first-line therapy’ usually implies the administration of SACT to patients with unresectable advanced cancer;^5^ the same term may, however, also be applied to patients receiving treatment for early cancer,^6^ those receiving such therapy in an effort to render the metastatic disease resectable,^7^ those receiving any initial systemic therapy (irrespective of cancer stage),^8^ and those receiving localised anti-cancer modalities such as radiotherapy^9^ or highly focused ultrasound.^10^ Importantly, the current use of LoT makes no reference to the total number of lines received by a given patient to date, which may be important in situations such as patients currently receiving ‘first-line therapy’ for metastatic breast cancer who may have previously received multiple LoTs as (neo)adjuvant therapy.

Defining and enumerating prior LoT is not straightforward. Patient pathways and records, whether electronic, paper or a mixture of the two, are often voluminous, making determination of the LoT time-consuming, resource-intensive and error-prone. Furthermore, as mentioned above, the implied meaning of the term ‘LoT’ can vary, depending on the type of cancer, the treatment intent, and definitions used in a given study protocol or treatment guideline. Principles for determining LoT have been proposed in patients with myeloma,^11^ and also in the specific context of claims-based retrospective studies,^12^ the latter, however, lacks sufficient clinical context or guidelines to be widely applied in routine practice or in clinical research. The UK Systemic Anti-Cancer Therapy dataset incorporates treatment ‘programmes’ and ‘regimens’, numbered according to their chronological order of commencement in the history of managing each patient’s treatment.^3^ However, to the best of our knowledge, no comprehensive guidelines exist for determining LoT across different solid cancers.

Several challenges exist to assigning LoT in a uniform way. The first of these is a lack of standard definitions and terminology. In addition, it is not always clear which therapies should be assigned a LoT. Furthermore, no standard format exists for expressing LoT in a way that would succinctly convey multifaceted, complex and clinically relevant information. There is, therefore, a lack of uniform methodology for enumerating LoT in patients with solid cancers and, in the absence of standard guidelines, clinicians and investigators often apply their own interpretation, resulting in variability and potential mis-classification,^13^ thereby hindering both clinical care and research.

In this article, we provide our perspective on a systematic and comprehensive framework to determine LoT in a uniform manner, such that it is applicable to most common clinical scenarios across solid malignancies. We begin by defining key terms that are required to determine the LoT (see Table 1). Next, we consider the complex issue of which therapies should constitute a LoT and how they should be characterised. Finally, we propose a novel and standardised format for reporting LoT.

**Table 1:** Definitions of key terms.

Term	Definition
Anti-cancer agent	A (bio)pharmaceutical product used for the treatment of malignant disease, including—but not limited to—cytotoxic, endocrine, targeted, immunotherapy (including vaccine and cell and gene therapy) and radiopharmaceuticals (e.g. radioisotopes, radio-labelled monoclonal antibodies, radioactive microspheres) that has been approved by the FDA and/or the EMA for treatment of any cancer. Note: (1) An anti-cancer agent in clinical development not yet approved by the FDA and/or EMA for treatment of any cancer (as on date of starting such therapy) should be termed an ‘experimental anti-cancer agent’; (2) Supportive therapy (e.g. haematopoietics, drugs for preventing skeletal related events, etc) should not be considered as an anti-cancer agent.
Anti-cancer modality	The type of therapy used to remove, kill or suppress cancer cells, including: • Surgery (therapeutic and including, but not limited to, open surgery, laparoscopic surgery, video-assisted thoracic surgery, robot-assisted surgery, etc.), but excluding diagnostic biopsy; • Radiotherapy (including, but not limited to, external beam radiation, brachytherapy, proton beam, stereotactic radiosurgery) but excluding radiopharmaceuticals (e.g. radioisotopes, radio-labelled monoclonal antibodies, radioactive microspheres); • Systemic anti-cancer therapy (SACT; see definition below); • Other (including, but not limited to, high-intensity focused ultrasound, cryotherapy, thermal ablation, photodynamic therapy, hyperthermia, vascular embolisation and anti-cancer agents not meeting the definition of SACT).
Clinical progression of disease (cPD)	The clear worsening of the patient’s clinical status or prognosis in the opinion of the treating clinician, taking into consideration clinical findings, imaging (including, but not limited to, objective imaging response criteria) and laboratory test results.
Clinical setting	The maximum extent of cancer spread experienced by the patient to date, denoted as A: Early setting (operable, without known distant metastasis); B: Locally advanced setting (inoperable, without known distant metastasis); C. Metastatic setting (operable/inoperable, with known distant metastases) Note: Operable implies that in the opinion of the clinician all known cancer can be completely removed surgically; Inoperable implies that in the opinion of the clinician all known cancer cannot be completely removed surgically.
Line of therapy (LoT)	A serial chronological number assigned to each systemic anti-cancer therapy (SACT) and experimental SACT administered to a patient and denotes a discrete attempt to treat the cancer. Note: LoT is reported in the format LoT N (CLoT + PLoT); CLoT is the number of SACT administered with curative intent and/or in the early setting (i.e. operable, without known distant metastasis); PLoT is the number of SACT given with palliative (i.e. non-curative/life-extending) intent and/or in the advanced setting (i.e. inoperable and/or with known distant metastases); N is the sum of CLoT and PLoT.
Systemic anti-cancer therapy (SACT)	SACT has the following features: • It consists of one or more anti-cancer agents or experimental anti-cancer agents, which can be administered alone or in combination or sequence (which might include alternating, hybrid, continuation maintenance therapy and/or switch-maintenance therapy); • It is prospectively planned; • It is usually (but not necessarily) administered in repeating cycles; • It is administered systemically, or via local/regional routes but with the intention of systemic effect or significant reduction of overall tumour burden in the opinion of the clinician; • It is given at a clinically relevant dose for a duration that is expected to exert systemic anti-cancer effect. Note: (1) If the SACT is composed exclusively of experimental anti-cancer agent(s), the prefix ‘Experimental’ should be added to such SACT; (2) A patient participating in a study where the control arm consists only of a placebo should not be considered to have received a SACT in that trial unless unblinded information is available; (3) If an FDA/EMA-approved anti-cancer agent is used with the intention of providing supportive/symptomatic care (e.g. dexamethasone is approved for multiple myeloma, but might also be used to treat nausea and vomiting in patients with other cancers), it should not be considered as an SACT.
Treatment intent	Treatment intent, in the opinion of the treating clinician, can be curative or palliative (i.e. non-curative/life-extending) (A) Curative therapy aims at complete elimination of cancer and preventing its recurrence; (B) Palliative therapy aims at improving the quality and/or quantity of life but without the expectation of cure.

Our proposed framework relies on many terms and concepts that are used only for solid cancers (such as ‘local/metastatic’, ‘operable/inoperable’, or ‘early/advanced’), and consequently is not applicable to haematological malignancies.

## A proposed systematic and comprehensive framework to determine LoT

### Terminology and definitions

In our definition of a LoT (see Table 1), a serial chronological number is assigned to each administered SACT that denotes a discrete attempt to treat the patient’s cancer. A SACT consists of one or more anti-cancer agents, and can include alternating,^14^ hybrid,^15^ continuation maintenance^16,17^ or switch-maintenance components.^18^ A SACT should be prospectively planned and well defined. For example, a pre-planned sequence of platinum-based chemotherapy followed by switch maintenance (which the National Comprehensive Cancer Network defines as “…the initiation of a different agent, not included as part of the first-line regimen, in the absence of disease progression, after 4–6 cycles of initial therapy”)^19^ should be considered a single SACT. Similarly, the pre-planned sequence of a conditioning/lympho-depleting chemotherapy regimen followed by CAR-T cell transfusion to a patient with synovial sarcoma constitutes a single SACT.^20^ As another example, the systemic anti-cancer agents embedded within a pre-planned sequence of neoadjuvant chemotherapy, HER2-targeted therapy, surgery, radiotherapy, and hormonal therapy for a patient with early breast cancer should together be counted as a single SACT (see Table 2).

**Table 2:** The proposed standard dataset (top row) comprises the minimum data to be collected regarding the current disease status (columns 1–4) and anti-cancer therapy (columns 5–9) that would help determine the line of therapy (LoT, column 10).

(1)	(2)	(3)	(4)	(5)	(6)	(7)	(8)	(9)	(10)
Serial No.	Clinical setting	Treatment intent	Date of most recent clinical progression of disease (cPD)	Anti-cancer modality	Start date	Stop date	Reason for stopping	Comments	Line of therapy N (CLoT + PLoT)
1	2A Early	3A Curative	NA (Date of diagnosis: 1999 Dec 20)	5A: i, ii SACT: Cytotoxic, Endocrine 5C**:** Surgery 5D**:** Radiotherapy	2000 Jan 01	2005 Aug 14	8B Completed	5FU + epirubicin 75 + cyclophosphamide x6 → breast conserving surgery → EBRT → tamoxifen x 2 yrs → letrozole x 3 yrs	1 (1 + 0)
2	2A Early	3A Curative	2010 Dec 22	5A: ii SACT: Endocrine 5C**:** Surgery	2011 Jan 01	2013 Jan 01	8B Completed	Isolated local recurrence, mastectomy, letrozole x 2 yrs	2 (2 + 0)
3	2B Locally advanced	3B Palliative	2015 Feb 01	5A: ii SACT: Endocrine	2015 Feb 07	2016 Sep 19	8A cPD	Anastrazole	3 (2 + 1)
4	2C Metastatic	3B Palliative	2016 Sep 15	5A: ii, iii SACT: Endocrine, Targeted	2016 Oct 02	2017 Jan 07	8A cPD	Everolimus + Exemestane	4 (2 + 2)
5	2C Metastatic	3B Palliative	2017 Jan 07	5A: i SACT: Cytotoxic	2017 Feb 01	2017 Jul 17	8C Toxicity	Paclitaxel (stopped due to neuropathy)	5 (2 + 3)
6	2C Metastatic	3B Palliative	2017 Jan 07	5A: i SACT: Cytotoxic	2017 Aug 09	2017 Dec 23	8A cPD	Docetaxel	5 (2 + 3)
7	2C Metastatic	3B Palliative	2017 Dec 17	5A: i SACT: Cytotoxic	2018 Jan 08	2018 Aug 15	8A cPD	Capecitabine	6 (2 + 4)
8	2C Metastatic	3B Palliative	2018 Aug 15	5A: i SACT: Cytotoxic	2018 Sep 01	2018 Dec 30	8A cPD	Eribulin	7 (2 + 5)
9	2C Metastatic	3B Palliative	2019 Jan 03	5A: ii SACT: Endocrine	2019 Jan 27	2019 Jun 15	8A cPD	Fulvestrant	8 (2 + 6)
10	2C Metastatic	3B Palliative	2019 Jun 04	5B: iii Experimental SACT: Targeted	2019 Jun 22	2020 Apr 30	8A cPD	Experimental agent (small molecule)	9 (2 + 7)

The table has been populated with an illustrative example of enumeration of the LoTs for a hypothetical patient with breast cancer who received multiple LoTs.

Dates should be reported in the format YYYY MMM DD, e.g. 2021 Jan 01.

LoT is recorded in the format LoT N (CLoT + PLoT); CLoT is the number of SACT administered with curative intent and/or in the early setting (i.e. operable, without known distant metastasis); PLoT is the number of systemic anti-cancer therapy (SACT) with palliative intent (i.e. non-curative/life-extending) and/or in the advanced setting (i.e. inoperable and/or with known distant metastases); N is the sum of CLoT and PLoT.

2A: Early setting (operable, without known distant metastasis); 2B: locally advanced setting (inoperable, without known distant metastasis); 2C: metastatic setting (operable/inoperable, with known distant metastases); 3A: curative; 3B: palliative; 5A: systemic anti-cancer therapy (SACT); 5B: experimental SACT (i.e. SACT without any FDA/EMA-approved anti-cancer agent); [5A and 5B are further categorised as: (i) cytotoxic; (ii) endocrine therapy; (iii) targeted; (iv) immunotherapy (including vaccine and cell and gene therapy); (v) other]; 5C: surgery; 5D: radiotherapy; 5E: other.; 8A: clinical progression of disease (cPD); 8B: completed planned anti-cancer therapy; 8C: toxicity; 8D: patient/clinician choice; 8E: death; 8F: other (e.g. financial or any other reason).

Although most anti-cancer agents are delivered orally or intravenously, other routes—such as intramuscular, subcutaneous, intrathecal, intraarterial, intraperitoneal, intravesical, intralesional, cutaneous or intradermal—are also used,^21^ and the distinction between the aim of local and systemic administration is not always clear cut. In many situations, the aim of locally administered anti-cancer agents is to generate a durable systemic clinical response, as with the endocrine agent fulvestrant, which is given intramuscularly in women with metastatic breast cancer. It is also increasingly recognised that intratumoural injection of immunotherapy can lead not just to local priming, but can also have abscopal effects on distant tumours.^22^ Conversely, in other clinical scenarios such as treatment of liver metastases,^23^ isolated limb sarcomas,^24^ and other cases, the clinician might use anti-cancer agents with the deliberate aim of obtaining a local/regional, rather than systemic, effect. The development of radiopharmaceuticals such as radioactive iodine-131, 177Lu-dotatate and yttrium-90 has, to an extent, also blurred the margins between systemic therapy and radiotherapy. Taking all these scenarios into account, we define SACT as either being administered systemically or by local/regional routes with the intention of inducing a systemic effect or significantly reducing overall tumour burden, in the opinion of the treating clinician.

Sometimes anti-cancer agents are administered at doses that are substantially lower than usual—for example, when given concomitantly with radiotherapy or as metronomic chemotherapy; such treatment is frequently ‘off-label’. On other occasions, patients might experience severe toxicity from a SACT so only a small cumulative dose is administered; an extreme example could be a patient experiencing an anaphylactic reaction, precluding further use. To qualify for assignment of a LoT, the treating clinician must be of the opinion that the patient has received the anti-cancer agent(s) at a clinically relevant dose and duration that could potentially exert an anti-cancer effect.

Clinical progression of disease (cPD) is a clear worsening of the patient’s clinical status or prognosis in the opinion of the treating clinician, taking into consideration clinical findings, imaging (including, but not limited to, objective imaging response criteria) and laboratory test results. While the clinician should align as far as possible with the definition of progressive disease (PD) by objective imaging response criteria,^25,26^ we propose that the term cPD be used in the scenario in which the treating clinician is of the opinion that a patient’s cancer is progressing but does not yet meet the criteria for PD by objective imaging response criteria, or if images are not available or evaluable. Conversely, a patient might technically fulfil the criteria for PD by objective imaging response criteria, but the clinician might be of the opinion that this is not clinically relevant—for example, if the ‘baseline’ scan was carried out some time before treatment started. The cPD designation is especially relevant when SACT is administered in routine practice in situations where objective imaging response criteria do not always play a key role in decision making. Within a clinical trial, cPD will usually equate to PD by objective imaging response criteria; however, a patient with stable disease by objective imaging criteria but ‘symptomatic deterioration’ would be designated as having cPD. This reflects routine practice, and is frequently accepted in clinical trials, but the cPD designation will, we hope, add clarity.

### Which treatments should be assigned a line of therapy?

Great heterogeneity exists in the literature and clinical trial protocols regarding what constitutes a LoT. Some clinical trial eligibility criteria allow prior systemic therapies for locally advanced inoperable cancer to be counted as a LoT,^27^ while others count SACT for distant metastases only;^28^ some criteria require completion of all curative systemic therapy,^29^ while others just require a certain number of prior SACTs irrespective of clinical setting or intent.^30,31^

**Fig. 1:**
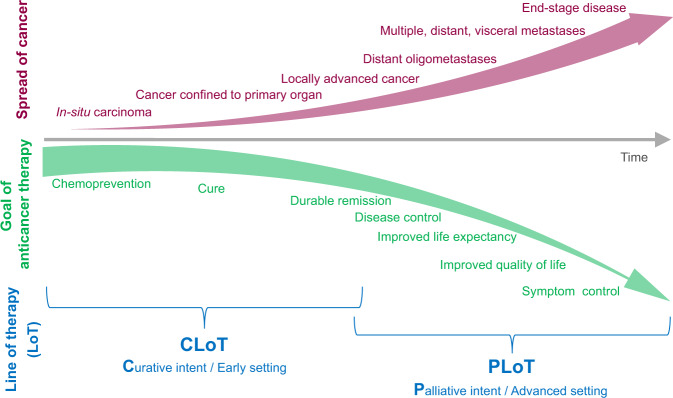
Over time, as a patient’s cancer burden increases, the corresponding goals evolve, and anti-cancer treatment is accordingly customised to provide goal-concordant care. Line of therapy (LoT) should be reported in a standard format as LoT N (CLoT + PLoT). The distinction between CLoT (curative intent and/or early setting) and PLoT (palliative intent and/or advanced setting) could be unclear in some scenarios, and the clinician should make a considered decision keeping in mind the type of cancer as well as individual patient characteristics.

Anti-cancer treatments are often tailored to the extent of disease, an approach termed ‘goal-concordant care’. For example, in patients with breast cancer, the therapeutic approach to early disease is distinct from that of metastatic disease, while therapy of patients with inoperable locally advanced disease is often similar to that of patients with disseminated malignancy.^2,32^ At present, many clinicians and clinical trial protocols assign LoT only to SACT for inoperable locally advanced or metastatic disease and/or when the treatment intent is palliative. There are two key arguments against this restrictive approach. First, the boundary between early and advanced disease settings, and that between curative and palliative intent, can be blurred. It is well-established that cancer is a systemic disease, with a spectrum of increasing cancer burden from small lesions in the primary organ to locally advanced carcinoma to oligometastatic disease to widespread visceral metastases (Fig. 1); the corresponding goals of anti-cancer therapy range from cure through achieving durable remission to symptomatic relief at different time points in a patient’s journey. Matching treatment intent to the extent of disease might be straightforward in some scenarios, such as curative intent in the early disease setting, but not in others. For example, the development of multiple lung metastases would make the intent of subsequent SACT palliative in a patient with breast cancer since advanced breast cancer is believed to be incurable;^2^ however, if the same clinical scenario of multiple lung metastases was seen in a patient with seminoma, the intent may still be curative,^33^ given the excellent chemosensitivity of this type of cancer. If a patient with a limited number of liver metastases receives preoperative chemotherapy followed by complete resection, some clinicians and trial protocols would not count this as a ‘line’ of SACT in the case of a colonic primary; by contrast, chemotherapy for treating liver metastases would count as a LoT for a patient with breast cancer. Hence, restricting LoT only to the advanced setting and/or when used with palliative intent could lead to inconsistencies among different solid cancers with regards to what should be counted as a ‘line’. Even within the same tumour type, such as breast cancer, therapy for locally advanced disease is counted as a LoT in some studies,^27^ but not in others.^28^ Second, there is evidence that the clinically meaningful benefit (such as objective response rate and progression-free survival) of subsequent SACTs decreases as the number of lines of prior SACT increases.^34–38^ It is reasonable to hypothesise that this inverse linear association should also apply to the total number of such SACTs administered, not just those given in the advanced/palliative setting. For example, patients with early breast cancer who have residual disease at surgery following neoadjuvant treatment,^39^ or who develop isolated locoregional recurrence,^40^ often receive additional systemic therapy that might well have an impact on subsequent therapeutic options and clinical outcomes, but such treatment is not currently counted as a LoT by many clinicians and trial protocols.

We propose that all SACTs, whether administered for early, locally advanced, or metastatic cancer, be assigned a LoT. It could be argued that local therapies including surgery, stereotactic ablative radiotherapy,^41^ brachytherapy, external beam radiotherapy, other methods of delivery of local energy (such as high-intensity focused ultrasound, cryotherapy, thermal ablation, photodynamic therapy, hyperthermia, etc.) and anti-cancer therapies that don’t currently meet the definition of SACT should also count as a LoT. For now, we propose that they not be included as their impact on subsequent systemic treatment choices is unclear, and to include them would add complexity.

If a patient develops primary cancer in more than one organ, the SACTs administered for each primary organ should be separately assigned LoTs. For patients who develop a second ipsilateral or contralateral cancer in paired organs such as the breast, the clinician should continue serial numbering of LoT if they consider it to be a locoregional recurrence from the initial cancer; alternatively, they should start a separate record if they consider it to be a new primary cancer.

### Proposed standardised format for reporting lines of therapy

As mentioned above, the current method of reporting LoT with a single ordinal number—for example, first-line metastatic—although simple and widely used, is often imprecise, liable to misinterpretation, and incomplete in capturing and expressing nuanced information about the number of prior therapies.

We propose that prior LoT should be reported in a standardised format as LoT N (CLoT + PLoT), where CLoT is the number of systemic regimens given with curative intent and/or in the early setting (that is, operable, with no known distant metastasis), PLoT is the number of systemic regimens administered with palliative (that is, non-curative/life-extending) intent and/or in the advanced setting (that is, inoperable and/or with known distant metastases), and N is the sum of CLoT and PLoT. See Table 2 for an illustrative example.

As previously discussed, the boundary between curative and palliative intent, and also between advanced and early setting, can sometimes be unclear, and the clinician should use their best judgement and dichotomise a given LoT into CLoT or PLoT, keeping in mind the type of cancer as well as individual patient characteristics. The intent of the treatment should take precedence in making this choice. In determining the LoT, the opinion of the treating clinician should usually prevail.

## Next steps

As a next step, multiple stakeholders from diverse backgrounds and regions, including clinicians subspecialised in different cancer types, informaticians, data scientists, patient advocates, regulatory authorities and representatives of cancer centres, clinical trial units, professional oncology societies, and contract research organisations, should discuss these proposals in detail, including the definition of standard dataset and guidelines, to facilitate their widespread adoption. In this section, we provide our views as a starting point for those discussions.

### Standardised dataset

We propose a standardised template (Table 2) for recording data that are critical for determining LoT. The adoption of such a standardised minimum dataset for collecting and annotating patient data would aid the uniform application of information regarding LoT. Most of these data are already present in different parts of the electronic health records (EHRs) and tabulating them chronologically in a standard format could facilitate the easier review of all SACT administered to a given patient.

The clinical setting and the treatment intent are required to dichotomise a given SACT into either CLoT or PLoT. The date of the most recent cPD is key for accurately enumerating LoT. Recording all anti-cancer therapies would allow the entire treatment history to be easily reviewed in chronological order. Additionally, the temporal sequence (e.g. chemotherapy followed by surgery) or combination of approaches (e.g. chemotherapy and radiation therapy) could provide insights into treatment intent. Likewise, the reason for stopping a given SACT is important when determining LoT.

The use of a standardised dataset would yield several advantages. First, it would allow us to identify patients for specific trials more easily. For example, potential participants for a study requiring patients who have received ‘…no more than two separate lines of treatment for metastatic colorectal cancer’ (irrespective of the treatment intent)^42^ could be identified by counting all SACTs where the clinical setting option ‘Metastatic (Operable/inoperable, with known distant metastases)’ has been selected as the option (see footnotes to Table 2). As another example, if a protocol states that prior SACT given in the early setting should be counted as having been given for advanced disease if the disease-free interval is of less than a specified duration,^43^ these patients could be identified from the interval between the stop date in a given row and date of cPD in the next row. This dataset, which summarises patient treatment in a single table, would help referrals by use of a familiar and standard template. Also, when a patient being treated at a hospital enters a clinical trial, the investigators could authorise their local EHR to directly auto-populate (under secure and compliant systems) the trial database with the redacted dataset related to prior lines of therapy, thus reducing effort and errors.

Importantly, this uniform characterisation of LoT would facilitate the auditing of SACT, which is an area of increasing interest. For example, in the UK, the SACT dataset collects systemic anti-cancer therapy prescriptions from all NHS England providers and aims to allow better understanding of treatment patterns and outcomes on a national scale.^3^ The UK database does not, however, characterise LoT as comprehensively as the current proposal would do.

Widespread consultation among key stakeholders should take place to establish a consensus on a standardised dataset that could make LoT determination easier, more uniform, and quicker, and subsequently embed it within hospital prescribing systems and EHRs. It might be possible to use such a standardised dataset in the form of an electronic spreadsheet with drop-down menus, in a form that can interface with other databases, or even in a paper format.

### Standardised guidelines to assess LoT

The development of standardised guidelines for enumerating LoT—applicable to all solid cancers—should be a key endeavour of the oncology community. Counting LoT is straightforward and non-controversial in many common clinical scenarios; draft guidelines for these are presented in Table 3. Developing broadly acceptable guidelines for some other clinical situations, such as handling unplanned changes in SACT in the absence of cPD, and if LoTs should be assigned to radiotherapy or surgery, could prove to be more difficult; such scenarios need to be discussed with key stakeholders in order to evolve a consensus. For example, if an anti-cancer agent is discontinued due to toxicity and substituted by another agent of a different class, should the same LoT be retained? Financial considerations can be important factor in cancer therapy,^44^ so if the use of a SACT is stopped for financial reasons only and substituted with another, should that be assigned a new LoT? If a perimenopausal woman attains biochemical menopause while on tamoxifen for metastatic breast cancer and her treatment is changed to an aromatase inhibitor in the absence of cPD or toxicity, should these be counted as separate lines? Should rechallenge^45,46^ or retreatment^47^ be assigned a new LoT when it is not part of a pre-planned alternating, hybrid, continuation maintenance or switch-maintenance therapeutic plan? What impact, if any, should a ‘drug holiday’ have on numbering subsequent LoT?

**Table 3:** Draft guidelines for determining lines of therapy in patients with solid cancers.

(1) Once clinical progression of disease (cPD) is documented, assign a new line of therapy (LoT) to the next systemic anti-cancer therapy (SACT).
(2) In the absence of cPD, if an anti-cancer agent that is part of a SACT is discontinued due to toxicity and substituted by another anti-cancer agent of the same class, retain the same LoT.
(3) In the absence of cPD, if one or more new anti-cancer agent is added to an ongoing SACT^a^ consider this a new SACT, and assign it a new LoT
(4) Irrespective of cPD, if one or more anti-cancer agent is discontinued from an ongoing SACT for any reason, retain the same LoT for the remaining anti-cancer agents.
(5) Irrespective of cPD, if the dose or schedule or route of administration of one or more anti-cancer agent of an ongoing SACT is modified for any reason, retain the same LoT.

^a^Except if prospectively planned per definition of SACT in Table 1, and except in scenario covered above in Guideline No. 2.

Discussions based on the Delphi method of consensus development and/or benchmarking studies should take place to establish agreement on a standardised set of comprehensive guidelines for LoT determination. Such broader testing for acceptability within the oncology community is necessary, and we propose to continue working in this direction, along with other experts and stakeholders.

## Conclusion and discussion

We propose a simple, comprehensive, and reproducible framework consisting of common definitions, standardised dataset, a novel recording format, and standard guidelines for determining LoT. This framework is applicable to the majority of commonly encountered clinical and research scenarios in patients with solid cancers and can be applied to prospective, contemporaneous or retrospective recording and determination of lines of prior anti-cancer therapies. This proposal should form the basis for wider discussions with a view to optimisation, validation and subsequent broad adoption.

The extraordinary diversity in the clinical manifestations of cancer as well as the tremendous complexity of its treatment pose huge challenges in formulating general criteria or guidelines that are applicable across all (or most) types of solid cancer; notable exceptions/successes include tumour staging (TNM), performance status (PS), the common terminology criteria for adverse events (CTCAE) and the response evaluation criteria in solid tumours (RECIST and iRECIST). It has, however, proved difficult to consistently and reproducibly enumerate the prior LoT administered to patients with cancer, for several reasons, including the complexity of therapy, which includes multiple phases and settings, as well as planned and unplanned gaps in its administration; to date, standard guidelines for determining LoT are available only for patients with multiple myeloma.^11^

Definitions of LoT vary widely, which has implications for trials and clinical practice. For example, an analysis by researchers from the University of Oxford of cancer drug appraisals by the UK National Institute for Health and Care Excellence highlighted that the population recruited to cancer trials does not always precisely match the licenced indication for the anti-cancer agent being evaluated, with disparities related to specific line of treatment as well as prior drug exposure, indication under consideration, specific mutation, and cancer type.^13^

A greater level of uniformity is valuable in improving the quality of any system, including complex health systems,^48^ and one way this can be achieved is by harmonising nomenclature, definitions and methodologies.^49–52^ In addition to benefits with relevance to clinical trials, regulatory approvals and re-imbursement, our proposed format for documenting LoT captures multifaceted data in a standardised manner. This could allow those data to be analysed in nuanced and clinically relevant ways, leading to the creation of searchable institutional databases that allow automated queries to identify specific patient populations for auditing or clinical trial participation, facilitating cross-trial comparison of data, and enabling more accurate meta-analyses.

The documentation relating to clinical practice and research in patients with cancer is already burdensome.^53^ Some data elements proposed in this article would need to be provided by the clinician who prescribed the anti-cancer therapy, or a suitably trained delegate such as a clinical trainee or oncology nurse. It is our hope that the proposed standard dataset and the uniform LoT N (CLoT + PLoT) format will ultimately reduce the overall burden by simplifying and standardising the collection of treatment and LoT data.

We acknowledge that our proposed framework for determining LoT does not, at this time, cover all possible clinical scenarios and that differences of opinion with some elements might arise. Considering the complex real-world challenges in healthcare and the heterogeneity of the information systems across and within countries, it remains to be seen how quickly our proposal is adopted at least in referral academic cancer centres, and how widely applicable it eventually proves to be.

In dealing with the enormous complexity of this subject, we were guided by Simon’s principle of ‘satisficing’; namely, that we can either find optimum solutions for a simplified world, or satisfactory solutions for a more realistic world.^54^ We have opted for the latter, which we judge appropriate given the ever-increasing complexity of cancer care. Remaining questions include whether locoregional treatments given with the intent of treating local disease alone, including radiotherapy and surgery, should also count towards LoT. Likewise, although we propose guidelines for enumerating LoT in most common scenarios, there remain issues for further discussion around assigning LoTs, especially around unplanned changes to SACT in the absence of cPD. Imminent next steps would include the commencement of broad-based discussion with colleagues, experts, and stakeholders to evolve a consensus on the standardised dataset and guidelines to be used for determining LoT, which could then be piloted in one or more cancer centres using retrospective chart review. Lessons learnt from this exercise could then be applied when evaluating the use of the guidelines in patients with a broader range of solid cancers in more diverse settings where cancer services and trials are delivered.

In the future, artificial intelligence (AI) solutions might be able to accurately determine LoT directly from EHRs. Machine-learning algorithms that are currently being developed to estimate the number of LoT mainly rely on drug names, dose, repetition, cadence and other such objective measures^8,55,56^ but these are not ready for prime time use. There is, therefore, a need for a practical solution that can be implemented at pace and scale. Moreover, AI solutions for LoT assignment would probably be accelerated by the adoption of the standard definitions, dataset, novel recording format, and guidelines that we propose.

## Data Availability

All data mentioned in this manuscript are from publicly available sources.
